# An Optimized Two-Step Magnetic Correction Strategy by Means of a Lagrange Multiplier Estimator with an Ellipsoid Constraint

**DOI:** 10.3390/s18103284

**Published:** 2018-09-29

**Authors:** Linlin Xia, Jingtong Geng, Hanrui Yang, Yunqi Wang, Zhaolong Fu, Bo Meng

**Affiliations:** 1 School of Automation Engineering, Northeast Electric Power University, Jilin 132012, China; jingtong0611@163.com (J.G.); yanghanrui1208@163.com (H.Y.); 2 School of Electrical and Information Engineering, The University of Sydney, Camperdown, NSW 2006, Australia; 4490980@gmail.com; 3 Department of Engineering Management, Shenzhen Yuhu Power Co., Ltd., Shenzhen 518111, China; fuzhaolong_0406@126.com; 4 School of Computer Science, Northeast Electric Power University, Jilin 132012, China; mengbo_nannan@163.com

**Keywords:** two-step optimized correction, ellipsoid constraint, non-deterministic magnetic interferences, L-M fitting, Lagrange multiplier estimator

## Abstract

The geomagnetic field is as fundamental a constituent of passive navigation as Earth’s gravity. In cases where no other external attitude reference is available, for the direct heading angle estimation by a typical magnetic compass, a two-step optimized correction algorithm is proposed to correct the model coefficients caused by hard and soft iron nearby. Specifically, in Step 1, a Levenberg-Marquardt (L-M) fitting estimator with an ellipsoid constraint is applied to solve the hard magnetic coefficients. In Step 2, a Lagrange multiplier estimator is used to deal with the soft magnetic iron circumstance. The essential attribute of “the two-step” lies in its eliminating the coupling effects of hard and soft magnetic fields, and their mutual interferences on the pure geomagnetic field. Under the conditions of non-deterministic magnetic interference sources with noise, the numerical simulation by referring to International Geomagnetic Reference Field (IGRF), and the laboratory tests based upon the turntable experiments with Honeywell HMR3000 compass (Honeywell, Morristown, NJ, USA) conducted, the experimental results indicate that, in the presence of the variation of multi-magnetic interferences, the RMSE (Root Mean Square Error) value of the estimated total magnetic flux density by the proposed two-step estimator falls to 0.125 μT from its initial 2.503 μT, and the mean values of the heading angle error estimates are less than 1°. The proposed solution therefore, exhibits ideal convergent properties, fairly meeting the accuracy requirements of non-tactical level navigation applications.

## 1. Introduction

Magnetic compasses are usually considered effective measuring units for heading angle estimates, the accuracy of which, in general, will determine the performances of the whole navigation system [1,2]. In practical measurements, the measured data derived from uncontrolled environmental magnetic fields will lead to large error in evaluating the relevant attitude parameters [3,4]. Among the error sources, hard iron (mainly refers to the permanent magnets, the magnetized iron or steel) and soft iron (mainly refers to the iron which is easily magnetized once being placed in a magnetic field environment, especially the pure iron) interferences around the carriers, are generally the main error sources for magnetic measuring results [5]. Therefore, before being put into use, it is essential to correct the orthogonal tri-axis measurements of magnetic compasses. The research on dealing with magnetic correction can be classified into three categories. One is to utilize the reference information derived from, for example, Global Positioning System (GPS), the inertial measurement units (IMUs), etc., to correct the direct measuring results of magnetic compasses [6]. One is to use the external devices to measure the orthogonal tri-axis attitude angles of magnetic compasses as assisted sources [7]. Another one is to fulfill the magnetic correction by a class of algorithm-based means, termed ellipsoid fitting solutions [8]. 

Note that, differing from the hardware-upgrading schemes stated in the first two categories, for the ellipsoid fitting solutions, there is no need to take into account the cost, sizes or power consumption of the external devices. Therefore, for practical applications it is relatively feasible to adopt the algorithm-based means that concentrates on algebraic ellipsoid fitting and magnetic coefficient estimates. Since the direct solutions involve complex coupling consequences, the preferred solution therefore, is intended to underpin progressive estimation in many domains such as simplifying the calculating iteration, eliminating the mutual coupling effects, and reducing the computational work [9,10,11]. As a typical instance, D. Gebre-egziabher [12] proposed a nonlinear estimation algorithm to calibrate the solid-state strapdown magnetometers, first putting forward a two-step model. In Step 1, they skillfully introduced a group of auxiliary variables to denote nine parameters in the typical ellipsoid constraint, linearizing an equation set for the nine parameter estimates rather than performing the direct calculation for the hard and soft magnetic coefficients. In Step 2, these nine parameters were solved algebraically. Experiments showed that this two step estimator outperformed an extended Kalman filter formulation [12,13]. 

Inspired by this, in this framework, a proposed two-step magnetic correction algorithm with fusing the L-M fitting concept and the Lagrange multiplier idea is expected to eliminate the negative influences the hard and soft iron cause. The essence of which consists in the algebraic coefficient estimates corresponding to an irregular ellipsoid to a normal sphere transform by two steps. In view of the analyses above, the optimized correction algorithm appears fairly reliable, being characterized by better independence of the coefficient estimates in the presence of mutual coupling effects between hard and soft magnetic field interferences. 

The outline of the remainder of the paper is as follows: in the following section, the preprocessing of the magnetic data (more precisely, signals) is first stated. In Section 3, the optimized correction for hard magnetic coefficients by the L-M fitting estimator is described, and the Lagrange multiplier method process is also detailed allowing for ideal precision of the soft magnetic coefficient estimates. The numerical simulation and laboratory tests are carried out in Section 4, which emphasizes the experimental assessment on geomagnetic field measurement error estimates extracted from a calibrated compass under the conditions of various non-deterministic interferences in terms of, location, sample point shape, form (hard or soft iron) and intensity. Section 5 presents the main conclusion of this investigation.

## 2. Preprocessing of Magnetic Signals

Affected by internal structural and external environmental magnetic field uncertainties, the magnetic compass measurements are subject to noise interferences. In view of the frequency domain, improving the signal-to-noise ratio (SNR) of raw signals appears to be a principal solution to high-precision navigation service on the basis of external sensors’ measurements [14]. In these frameworks, our work mainly consists of three parts:

Step 1: We adopted the empirical mode decomposition (EMD) method to reduce the noise and filter the raw signals [15,16], and we obtained a series of multiple intrinsic mode function (IMF) components of the measured signals;
Step 2: For the noisy IMF components, we adopted the improved wavelet threshold denoising method (WTD) for signal noise reduction, and this process can be seen as completely retaining the pure IMF components;
Step 3: We further reconstructed a series of desired signals by fusing the original pure IMF components and the de-noised IMF components.

Since our experiments involve numerical simulation and actual laboratory tests, we emphasize that, for the generated data (the simulated magnetic flux density data), the Gaussian white noise is successfully eliminated by the above EMD theory. Analogously, in the real-time laboratory measuring situations, the smoothing requirements are also fully satisfied. 

## 3. Two-Step Optimized Magnetic Coefficients Correction Algorithm

When tilting a magnetic compass in any direction, observation shows that the shape of trajectory derived from orthogonal tri-axis outputs with respect to carrier coordinate reference maintains a normal sphere, which is characterized by the hypothesis that the magnetic compass is working in a pure geomagnetic environment without any interference like hard or soft iron on the host platform [17,18]. In practice, however, even though the intrinsic instrumentation errors of the magnetic compass, for example, scale factors, non-orthogonality, and offsets, exert certain influences on measuring results, we concentrate more upon eliminating the impacts of environmental magnetic field interferences, because in cases where hard or soft iron exists, we can only obtain an irregular ellipsoid rather than an approximate normal sphere [19,20,21]. The following subsections describe the elaborate tri-axis distribution transformation by mathematical means.

### 3.1. 1st Step Correction for Hard Magnetic Coefficients with L-M Fitting Estimator

Let the carrier coordinate reference be built along three axes of the magnetic compass. The hard and soft iron interference matrixes are, respectively, denoted as *H_i_* and $\varepsilon_{3\times 3}$ [22]. With real and measuring value matrix of geomagnetic field being denoted by *H_e_* and *H_m_* respectively, the error compensation model of compass is obtained as follows [23]:

(1) $$H_{e}=\varepsilon_{3\times 3}^{-1}\left(H_{m}-H_{i}\right)$$

Let $K=\varepsilon_{3\times 3}^{-1}$, rearranging Equation (1):

(2) $$H_{e}^{T}H_{e}={\left\|H_{e}\right\|}^{2}={\left(H_{m}-H_{i}\right)}^{T}K^{T}\left(H_{m}-H_{i}\right)$$

Let $M=K^{T}K/||H_{e}|{|}^{2}$, then Equation (2) can be further written as:

(3) $${\left(H_{m}-H_{i}\right)}^{T}M\left(H_{m}-H_{i}\right)=1$$

Since matrix *M* satisfies the Jo Riski decomposition [24], so *M* is symmetric and it follows that:

(4) $$M=\left[\begin{array}{ccc} a & b/2 & d/2\\ b/2 & c & e/2\\ d/2 & e/2 & f \end{array}\right]$$

where *a*, *b*,…, *f* denote the unknown elements of matrix *M*. On the assumption of $H_{i}={\left[\begin{array}{ccc} m & n & t \end{array}\right]}^{T}$, and $H_{m}={\left[\begin{array}{ccc} x & y & z \end{array}\right]}^{T}$, Equation (3), therefore, satisfies the following expression:

(5) $$a{\left(x-m\right)}^{2}+b(x-m)(y-n)+c{\left(y-n\right)}^{2}+d(x-m)(z-t)+e(y-n)(z-t)+f{\left(z-t\right)}^{2}=1$$

According to Equation (5), for *n* sets of *H_m_*, we get *n* linear equations in terms of the form below:

(6) $$\begin{array}{lll} F(X,k) & =z^{2}= & k_{\left(1\right)}x^{2}+k_{\left(2\right)}x+k_{\left(3\right)}y^{2}+k_{\left(4\right)}y+\\ & & k_{\left(5\right)}z+k_{\left(6\right)}xy+k_{\left(7\right)}xz+k_{\left(8\right)}yz+k_{\left(9\right)}\\ & =kX & \end{array}$$

where $\begin{array}{l} k=[\begin{array}{cccc} \begin{array}{cc} -\frac{a}{f} & \frac{2am+bn+dt}{f} \end{array} & -\frac{c}{f} & \frac{bm+2cn+et}{f} & \frac{dm+en+2ft}{f} \end{array}\\ \begin{array}{cccc} -\frac{b}{f} & -\frac{d}{f} & -\frac{e}{f} & -\frac{\left(am^{2}+\mathrm{bmn}+cn^{2}+\mathrm{dmt}+\mathrm{ent}+{\mathrm{ft}}^{2}-1\right)}{f}] \end{array} \end{array}$, $X={\left[\begin{array}{ccccccccc} x^{2} & x & y^{2} & y & z & xy & xz & yz & 1 \end{array}\right]}^{T}$.

Let $k_{\left(i\right)}$ (*i* = 1, 2, …, 9) indicate the elements of known vector *k* (being evaluated beforehand by L-M fitting method), the hard iron interference matrix $H_{i}={\left[\begin{array}{ccc} m & n & t \end{array}\right]}^{T}$ with respect to *k*, therefore, can be calculated from the ternary equations set below:

(7) $$\{\begin{array}{c} k_{\left(2\right)}=-2k_{\left(1\right)}m-k_{\left(6\right)}n-k_{\left(7\right)}t\\ k_{\left(4\right)}=-k_{\left(6\right)}m-2k_{\left(3\right)}n-k_{\left(8\right)}t\\ k_{\left(5\right)}=-k_{\left(7\right)}m-2k_{\left(8\right)}n+2t \end{array}$$

### 3.2. 2nd Step Correction for Soft Magnetic Coefficients with Lagrange Multiplier Estimator

Let $X^{\prime }=H_{m}-H_{i}=\left[x^{\prime }\;y^{\prime }\;z^{\prime }\right]$ being a collection of data points after hard iron interference compensation, so that Equation (3) reduces to:

(8) $$X^{\prime }MX^{\prime }{}^{T}=1$$

Equation (8) in this case can be rewritten by substituting Equation (4), it follows that:

(9) $$a{x^{\prime }}^{2}+c{y^{\prime }}^{2}+f{y^{\prime }}^{2}+bx^{\prime }y^{\prime }+dx^{\prime }z^{\prime }+ey^{\prime }y^{\prime }=1$$

Equation (9) represents an ellipsoid with the following constraint:

(10) $$qJ-I^{2}=1$$

where, I≡a+c+f, $J\equiv ac+cf+af-b^{2}-d^{2}-e^{2}$, *q* is a unknown positive integer.

For each $X^{\prime }=\left[x^{\prime }\;y^{\prime }\;z^{\prime }\right]$, define a new vector in augmented form, $X_{i}{}^{\prime \prime }={\left[\begin{array}{ccccccc} {x^{\prime }}_{i}{}^{2} & {y^{\prime }}_{i}{}^{2} & {z^{\prime }}_{i}{}^{2} & {x^{\prime }}_{i}{y^{\prime }}_{i} & {x^{\prime }}_{i}{z^{\prime }}_{i} & {y^{\prime }}_{i}{z^{\prime }}_{i} & 1 \end{array}\right]}^{T}$, where *i* = 1, 2, …, *N* representing the measuring set number.

To estimate the coefficients *a*, *b*, *c*, *d*, *e* and *f* in Equation (9), let $v\equiv {\left[\begin{array}{cccc} \begin{array}{ccc} \begin{array}{cc} a & c \end{array} & f & b \end{array} & d & e & -1 \end{array}\right]}^{T}$ be a coefficient vector in general augmented form, whose values are to be determined.

Thus, rewrite Equation (9) in the following matrix form, and clearly it can be expanded to a set of linear equations:

(11) $$D^{T}v=0$$

where *D* = [ *X_1__′_*’ *X_2__′_*’* … X_i_*’’]$\in R^{7\times N}$, N≥10. Denote the geometric distance (the minimum distance between the measured $X^{\prime }$ and the optimal fitted ellipsoid) by the matrix below:

(12) $$\Psi ={\left\|Dv\right\|}^{2}=v^{T}D^{T}vD$$

Minimizing Equation (12) under the constraint indicated in Equation (10) is our direct objective. To achieve this, rewrite Equation (10) in terms of $v^{T}Cv=1$, and we get:

(13) $$C=\left[\begin{array}{ccccccc} -1 & \frac{q}{2}-1 & \frac{q}{2}-1 & 0 & 0 & 0 & 0\\ \frac{q}{2}-1 & -1 & \frac{q}{2}-1 & 0 & 0 & 0 & 0\\ \frac{q}{2}-1 & \frac{q}{2}-1 & -1 & 0 & 0 & 0 & 0\\ 0 & 0 & 0 & -q & 0 & 0 & 0\\ 0 & 0 & 0 & 0 & -q & 0 & 0\\ 0 & 0 & 0 & 0 & 0 & -q & 0\\ 0 & 0 & 0 & 0 & 0 & 0 & 0 \end{array}\right]$$

The robust solution to the above optimization problem, therefore, turns to a matter for skilled mathematic iteration.

Construct the simultaneous equations by typical Lagrange multiplier model:

(14) $$L\left(v,\lambda \right)=\Psi -\lambda \left(v^{T}Cv-1\right)$$

where λ is the scalar Lagrange multiplier, and L represents “Lagrange function of”.

Differentiate the Lagrange function L with respective of v and λ respectively, and let ∂L/∂v=0 and ∂L/∂λ=0, we obtain:

(15) $$DD^{T}v=\lambda Cv$$

(16) $$v^{T}Cv=1$$

Note here, Equation (15) has only one solution when *q* > 3, which denotes the general eigenvector associated with the unique positive eigenvalue of a generalized eigenvalue equation $DD^{T}v=\lambda Cv$.

Solving for Equations (15) and (16), we reconstruct $DD^{T}$ and v in the following forms:

(17) $$DD^{T}=\left(\begin{array}{cc} P_{11} & P_{12}\\ P_{12}^{T} & P_{22} \end{array}\right)$$

(18) $$v=\left(\begin{array}{c} v_{1}\\ v_{2} \end{array}\right)$$

where, the matrixes *P*_11_, *P*_12_, *P*_22_ are of size 6 × 6, 6 × 1, and 1 × 1 respectively. Clearly, $v_{1}={\left[a\;c\;f\;b\;d\;e\right]}^{T}$,$v_{2}=-1$.

Hence we are dealing with an eigensystem of two equations given by:

(19) $$\left(P_{11}-\lambda C_{1}\right)v_{1}+P_{12}v_{2}=0$$

(20) $$P_{12}^{T}v_{1}+P_{22}v_{2}=0$$

where, $C_{1}\in R^{6\times 6}$ extracting the elements located in the first 6th row and 6th column of original matrix C. In Equation (20), *P*_22_ would be nonsingular in case of the relevant sampling points being not coplanar (since groups of $X^{\prime }$ data are derived from a 3-dimensional ellipsoid), then it can be verified that Equation (20) is solvable, which yields:

(21) $$v_{2}=-P_{22}^{-1}P_{12}^{T}v_{1}$$

Substituting Equation (21) into Equation (19), we obtain the generalized eigenvalue system equation [25], it follows that:

(22) $$C_{1}{}^{-1}\left(P_{11}-P_{12}P_{22}^{-1}P_{12}^{T}\right)v_{1}=\lambda v_{1}$$

As stated above, q is no less than 3. When q=4, $4J-I^{2}>0$, then the quadrique indicated by Equation (5) must be an ellipsoid, we say that is the sufficient condition for the present ellipsoid. In this case, we need to determine a bigger integer value q, so as to obtain a wider range of ellipsoid quadrique and satisfy $qJ-I^{2}>0$.

A rule of thumb, satisfactory in simple cases, is to designate q big enough (like 50), then we locate q by means of bisecting, viz., iteratively calculating it in the intervals between q and q/10.

**Figure 1:**
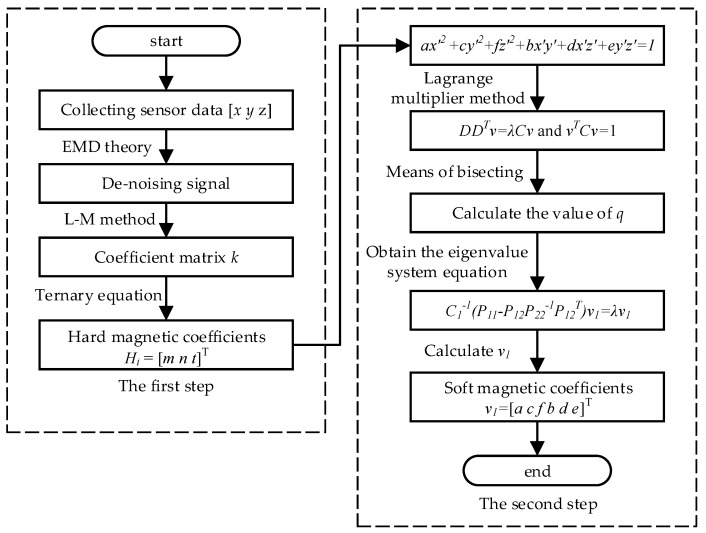
Algorithm flow chart for the geomagnetic measurement error correction.

The algorithm flow chart for the geomagnetic measurement error correction is shown in Figure 1. As indicated, the implementing volume has been divided into two parts, and the coefficients estimation appears to be merely forward, by means of which, we say the coupling effects’ elimination experienced by hard and soft magnetic field interferences is fairly achievable. For detailed experiments and result analyses, please refer to Section 4.

## 4. Experiments

Experimental observations consist of numerical simulations and laboratory tests. We are generally considering the simulation environment as the ideal geomagnetic field [26], since the pure geomagnetic field never exists under normal conditions of sequent tests.

### 4.1. Numerical Simualtions and Analyses

The geomagnetic field of the Jilin area (Jilin City, China) is designated as our simulation environment and the geomagnetic field distribution information is derived from the International Geomagnetic Reference Field (IGRF 12) by reference, with altitude 183.4 m in the urban area, latitude 43°52′ N, longitude 126°33′ E, and date June 2018 [27]. Two sets of values for the soft magnetic coefficient matrix $\varepsilon_{3\times 3}$ and hard magnetic coefficient matrix *H_i_* are designated, as denoted by HH (benchmark value) in Table 1. Similarly, the standard deviation of the white noise σ (μT) is set to be 0.1 and 0.01, respectively.

**Figure 2:**
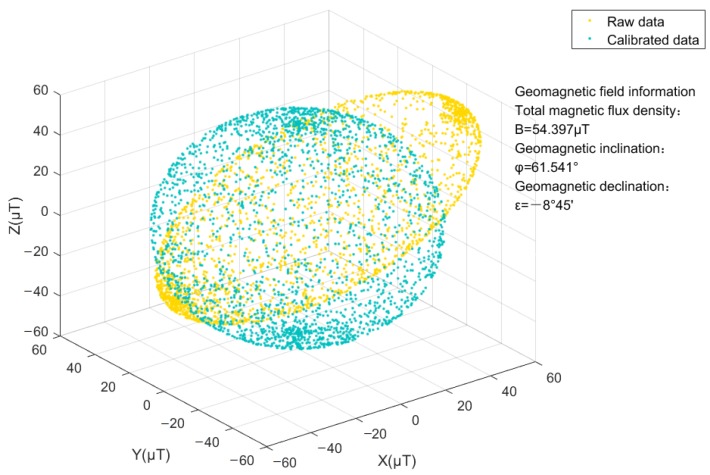
Tri-axis magnetic field distribution.

The simulated magnetic flux density in μTesla is derived from a Matlab simulation platform, and the preprocessing work focuses on smoothing the white noise involved. On the basis of the de-noising data, the orthogonal tri-axis magnetic field distribution before and after correction is illustrated by the dots in Figure 2, it is actually easier to indicate that the shape of dots changes from an irregular ellipsoid to an approximate normal sphere.

**Figure 3:**
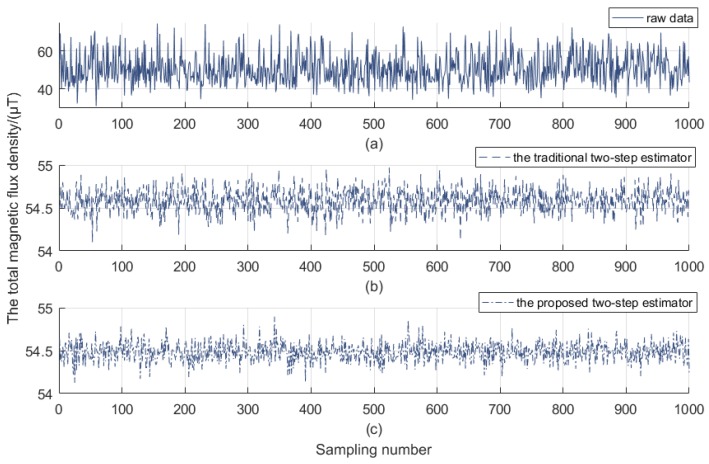
Total magnetic flux density before and after correction. (**a**) the error with raw data; (**b**) the result by the traditional two-step estimator; (**c**) the result by the proposed two-step estimator.

Figure 3 shows the error of total magnetic flux density. As presented, the error with raw data is very large as predicted (see Figure 3a), and the result (0.7 μT) by the proposed two-step estimator (see Figure 3c) exhibits better than that (1 μT) by the traditional two-step estimator stated in Reference [12,13] (see Figure 3b). It should be noted that the average of the total magnetic flux density conforms with the pre-set condition reference, i.e., *B*=54.397 μT (see Figure 2), and the solution adopted has been proved to possess strong convergence.

More detailed simulation results are given in Table 1, the derived *q* is based on a typical bisecting iteration. HH_1_ represents the estimated hard and soft magnetic coefficients, here [a] and [b] respectively represent the results by the proposed two-step estimator and the traditional two-step estimator, HP (μT) represents the maximum error of total magnetic flux density, HM (°) represents the mean of the heading angle error and HT (s) represents the average running time for the numerical simulation under the same conditions. The simulation results show that the soft and hard magnetic coefficients derived by the L-M fitting and the Lagrange multiplier estimator are much closer to the true values. In contrast, the optimized algorithm-based scheme shortened the executing time, leading to better heading angle estimation accuracy without implementing any hardware-upgrading means or integrating any external measurement units.

### 4.2. Laboratory Tests and Evaluations

**Figure 4:**
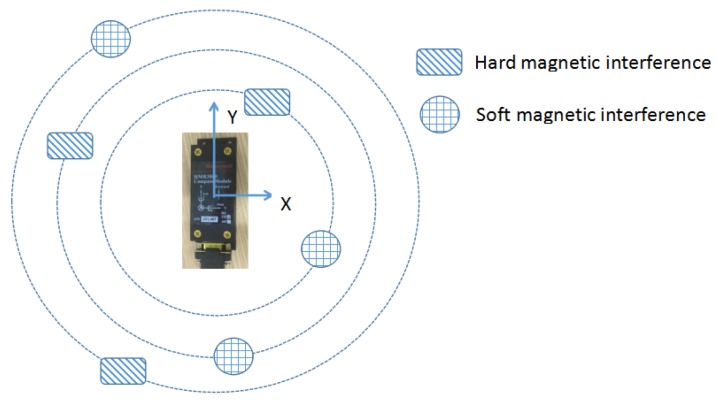
Interference source distribution.

The correction is experimentally assessed to evaluate the effectiveness of the optimized solution in practice. The laboratory tests are conducted based upon the turntable experiments with Honeywell HMR3000 compass (which measurement accuracy is within 100 nT) and the real geomagnetic field reference is provided by a high-precision proton magnetometer (the measurement accuracy is within 1 nT). To begin with, the compass is mounted on a stationary base on a two-axis horizontal turntable. In sequence, in the course of collecting orthogonal 3-axis magnetic flux density data, the compass rotates at a constant speed to capture uniformly distributed samples across different directions. Three experiments are implemented indicating different locations of interference sources, as illustrated in Figure 4. Specifically, it is to ensure that the hard and soft magnetic material be distributed around the magnetic compass in concentric circles. Referring to Reference [28], the radii of three interference sources are set to be 90, 70 and 50 cm, respectively.

**Figure 5:**
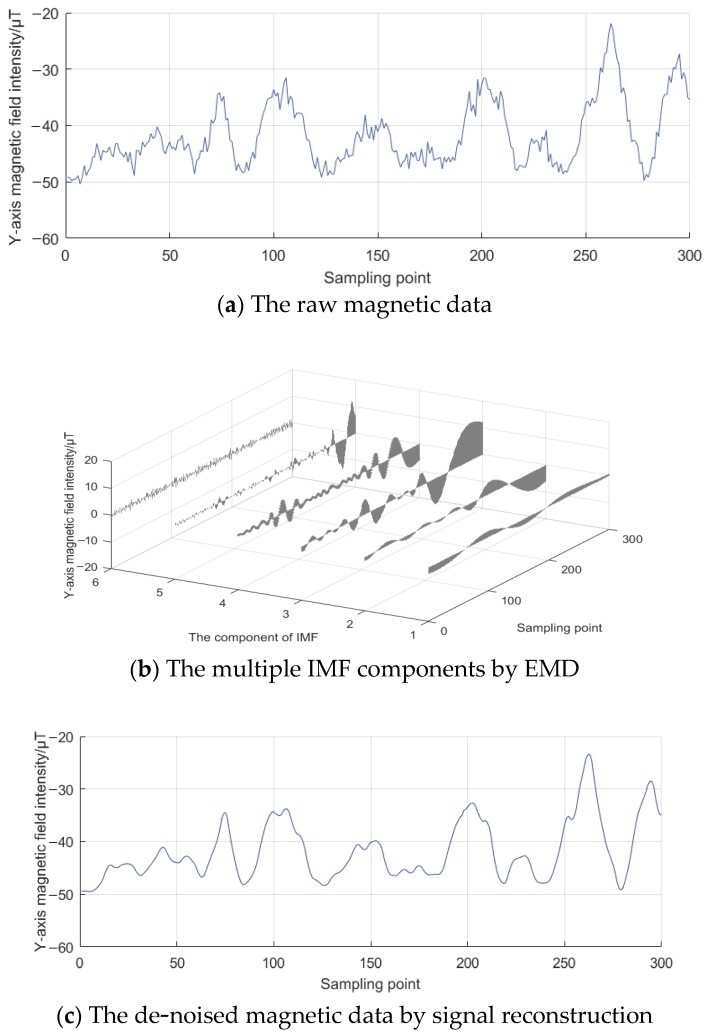
The preprocessing of raw measuring magnetic data.

As in the numerical simulation, the preprocessing of raw magnetic data should first be examined. Taking *Y*-axis measurements as an example, Figure 5a presents raw magnetic data derived from the Honeywell HMR3000 compass. Since the raw magnetic data contains noise, in order to filter the noise, the raw data was decomposed into six IMF components by the EMD method, diagrammatically represented by Figure 5b. In sequence, denoising the first three IMF components by improved WTD and fusing them with the remaining three IMF components, we achieved the final de-noised magnetic data. Figure 5c indicates the de-noised magnetic data by signal reconstruction in the frequency domain. Clearly, the de-noised data appears smoother, being better suited and favorable for the following ellipsoid fitting and relevant magnetic coefficients estimation.

**Figure 6:**
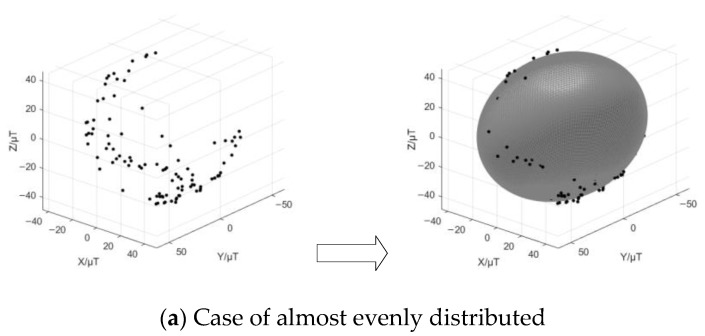

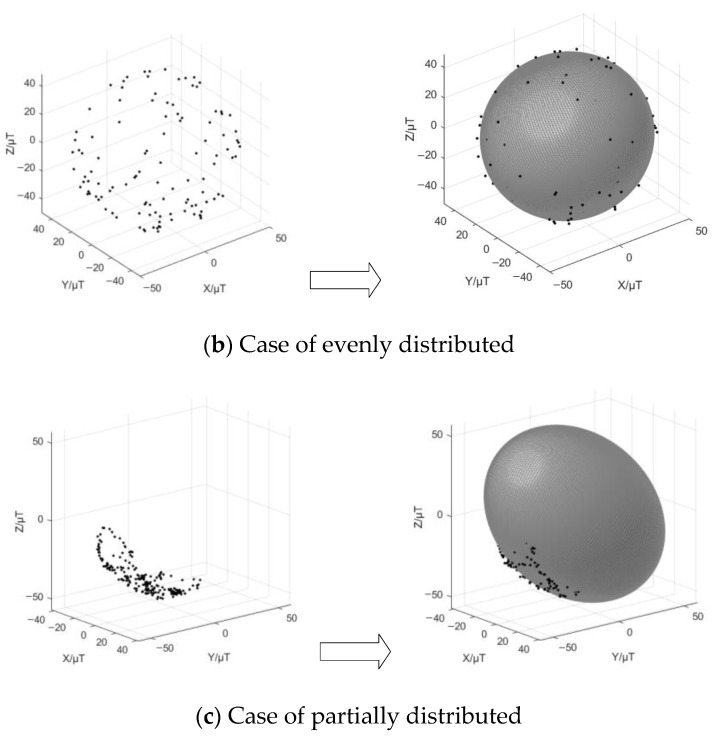
The orthogonal tri-axis magnetic field distribution and the fitted ellipsoid.

Figure 6 shows the fitted ellipsoids in cases of different circumstances where the orthogonal tri-axis magnetic field intensity is almost evenly distributed, evenly distributed, and partially distributed. Obviously, even the geomagnetic field distribution is more likely to form a regular ellipsoid, leading to an approximate normal sphere. In practice, however, it may well be that the turntable fails to ideally rotate at a constant speed, and the measured data consequently covers a very small portion of the ellipsoid surface. 

In this case, determining the accurate radian and radial length at the very start is of great importance, which is achieved through multiple iterations of *q*. In our framework, the proposed two-step optimized algorithm by the L-M fitting and the Lagrange multiplier estimator has been applied to a vastly wider range of datasets, and the fitting results are adequately satisfied.

**Figure 7:**
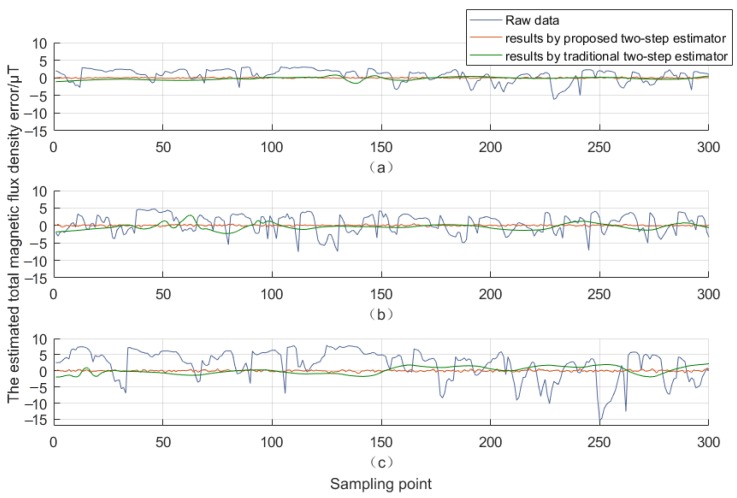
The estimated total magnetic flux density error. (**a**) the interference source radius is 90 cm; (**b**) the interference source radius is 70 cm; (**c**) the interference source radius is 50 cm.

The estimated total geomagnetic field measurement error is diagrammatically represented in Figure 7, with a-c corresponding to different interference sources nearby, referring to Figure 4, the radii of the interference sources appear to be 90 cm, 70 cm and 50 cm respectively. Clearly, the traditional two-step estimator reveals its limited capacity in more accurate error estimation, and the undesired curve wave also illustrates degrees of instability. The algorithm stability still needs to be enhanced. By comparison, the proposed two-step estimator presents the optimal error estimation irrespective of the variation of the environmental magnetic fields, whose results exhibit better convergent properties with respect to the coupling effects experienced by hard and soft iron.

**Figure 8:**
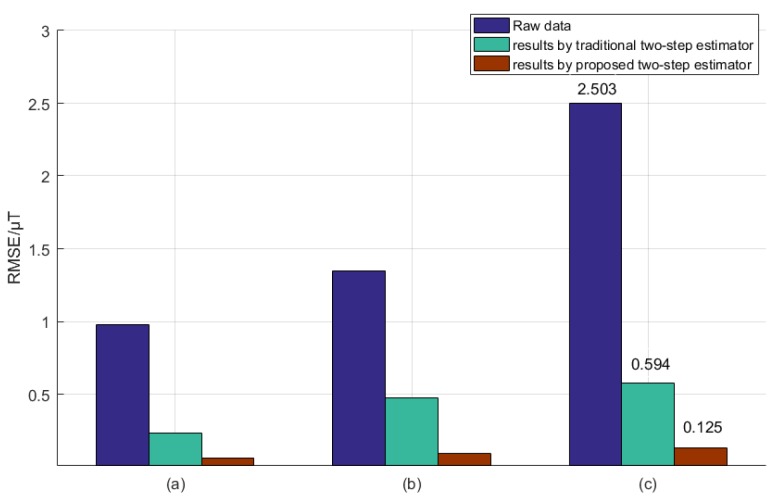
The comparison of RMSE. (**a**) the interference source radius is 90 cm; (**b**) the interference source radius is 70 cm; (**c**) the interference source radius is 50 cm.

The corresponding stochastic data (root mean square error, RMSE) comparison is illustrated by the bar graph in Figure 8. As shown, for the case that the hard and soft iron are very close to the magnetic compasses, the RMSE value of the estimated total magnetic flux density extracted from the proposed two-step estimator falls to 0.125 μT from the initial 2.503 μT (with raw data from Honeywell HMR3000). Specifically, the traditional two-step estimator presents a relatively large RMSE, and this is due to the fact that in step 2, in order to calculate the relevant nine parameters algebraically, a number of multivariate equations are involved with hardly guaranteeing the independence of the equations, that is, the solutions for nine parameters would be not unique, and thus the magnetic flux density error would be large. In contrast, even though the proposed two-step estimator follows the same line as the traditional one in the two-step magnetic correction design, the optimized estimator addresses this problem by means of multiple iterations of the equations involved, and after these proactive iterations, the calculation error decreases, leading to more ideal RMSE results. 

**Figure 9:**
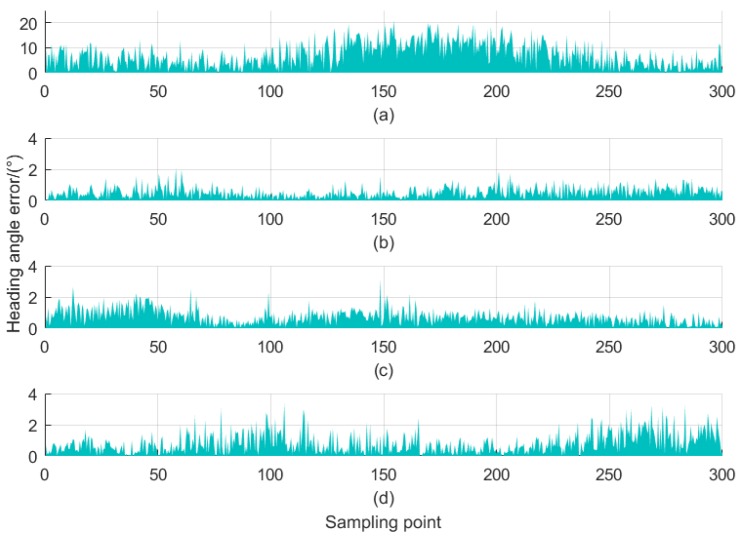
The estimated heading angle error before and after correction by the proposed two-step estimator. (**a**) before correction; (**b**) the interference source radius is 90 cm; (**c**) the interference source radius is 70 cm; (**d**) the interference source radius is 50 cm.

The estimated heading angle error before and after correction is presented in Figure 9. Clearly, whose peak values even approach 20° when directly using the raw data from Honeywell HMR3000, i.e., the measured data is not corrected at all (see Figure 9a). It should be noted that, in this case, the distance of the interference object nearby is set to be 90 cm, indicating that the interferences are not too severe. Through Matlab simulative calculations, the mean values of the estimated heading angle error corresponding to Figure 9b–d (whose radii of interference sources are 90, 70 and 50 cm, respectively) are 0.486°, 0.825° and 0.918°, respectively. It is obvious to indicate that, after the magnetic correction, the mean values of the heading angle error estimates are no more than 1° regardless of the difference of locations of interference sources and radii of concentric circles, and that the optimized two-step magnetic correction strategy fairly meets the accuracy requirements of non-tactical level navigation applications.

## 5. Conclusions

An optimized two-step model by the L-M fitting and the Lagrange multiplier estimator for magnetic field measurement correction was employed, after exhibiting ideal convergent properties in the numerical simulations, which was experimentally assessed with the Honeywell HMR3000 compass to eliminate the magnetic interferences caused by surrounding hard and soft iron. It demonstrated that, compared with the traditional two-step estimator, the proposed estimator effectively reduces the environmental magnetic field disturbance and its accuracy and reliability remain optimal with the variation of non-deterministic interferences. The experimental results indicate that the mean values of the heading angle error estimates are less than 1° under strong magnetic interferences. The algorithm-based solution with no extra assistant measurement units, therefore, may be considered as a reference tool for a class of magnetic field coefficients correction with environmental interferences hypothesis.

## Figures and Tables

**Table 1:** Simulation results.

**HH**	$\varepsilon_{3\times 3}$	$\left[\begin{array}{ccc} 0.83574 & -0.04635 & 0.02656\\ 0 & 1.12564 & 0.01152\\ 0 & 0 & 1.10233 \end{array}\right]$		$\left[\begin{array}{ccc} 0.93552 & 0.09654 & 0.05234\\ 0 & 1.14621 & 0.03207\\ 0 & 0 & 1.19657 \end{array}\right]$	
	*H_i_*	$\left[\begin{array}{ccc} 0.9 & 0.7 & -0.4 \end{array}\right]$		$\left[\begin{array}{ccc} 0.3 & -0.3 & 0.8 \end{array}\right]$	
σ(**μT**)		0.1	0.01	0.1	0.01
***q***		7	8	9	9
**HH_1_**	[a] *H_i_*	$\left[\begin{array}{ccc} 0.9007 & 0.6986 & -0.3990 \end{array}\right]$	$\left[\begin{array}{ccc} 0.9003 & 0.6988 & -0.4010 \end{array}\right]$	$\left[\begin{array}{ccc} 0.2991 & -0.3008 & 0.7983 \end{array}\right]$	$\left[\begin{array}{ccc} 0.2992 & -0.2998 & 0.8006 \end{array}\right]$
	[b] *H_i_*	$\left[\begin{array}{ccc} 0.9035 & 0.6162 & -0.3780 \end{array}\right]$	$\left[\begin{array}{ccc} 0.9044 & 0.6861 & -0.3941 \end{array}\right]$	$\left[\begin{array}{ccc} 0.3035 & -0.3062 & 0.9092 \end{array}\right]$	$\left[\begin{array}{ccc} 0.2150 & -0.3101 & 0.7390 \end{array}\right]$
	[a] $\varepsilon_{3\times 3}$	$\left[\begin{array}{ccc} 0.83601 & -0.04602 & 0.02675\\ 0 & 1.12537 & 0.01180\\ 0 & 0 & 1.10221 \end{array}\right]$	$\left[\begin{array}{ccc} 0.83594 & -0.04611 & 0.02678\\ 0 & 1.12541 & 0.01175\\ 0 & 0 & 1.10229 \end{array}\right]$	$\left[\begin{array}{ccc} 0.93596 & 0.09612 & 0.05265\\ 0 & 1.14602 & 0.03176\\ 0 & 0 & 1.19640 \end{array}\right]$	$\left[\begin{array}{ccc} 0.93601 & 0.09604 & 0.05254\\ 0 & 1.14592 & 0.03169\\ 0 & 0 & 1.19636 \end{array}\right]$
	[b] $\varepsilon_{3\times 3}$	$\left[\begin{array}{ccc} 0.83635 & -0.04711 & 0.02696\\ 0 & 1.12593 & 0.01211\\ 0 & 0 & 1.10286 \end{array}\right]$	$\left[\begin{array}{ccc} 0.83629 & -0.04705 & 0.02695\\ 0 & 1.12589 & 0.01208\\ 0 & 0 & 1.10281 \end{array}\right]$	$\left[\begin{array}{ccc} 0.93625 & 0.09787 & 0.05156\\ 0 & 1.14561 & 0.03286\\ 0 & 0 & 1.19689 \end{array}\right]$	$\left[\begin{array}{ccc} 0.93606 & 0.09721 & 0.05161\\ 0 & 1.14560 & 0.03279\\ 0 & 0 & 1.19687 \end{array}\right]$
**HP**(**μT**)	[a]	0.847	0.074	0.831	0.092
	[b]	0.941	0.085	0.970	0.183
**HM**(**°**)	[a]	0.656	0.230	0.696	0.247
	[b]	0.764	0.315	0.765	0.317
**HT**(**s**)	[a]	1.05	1.02	1.09	0.99
	[b]	1.42	1.35	1.44	1.39
